# Liquor Taraxaci

**Published:** 1843-01-21

**Authors:** 


### LIQUOR TARAXACI.

A very elegant preparation has been introduced under the above title, and which, from the strong taste it possesses of the recent root, has been much used by medical men who have confidence in the remedial power of Dandelion. The following formula has been communicated to us:—

Dandelion roots, perfectly clean, *dried*, and sliced, 18 ounces.

Infuse for 24 hours in a sufficient quantity of cold distilled water to cover them.

Press and set aside, that the feculæ may subside; decant and heat the clear liquor to 180° F., so as to coagulate the albumen; filter the liquid whilst hot, and evaporate in a dry room, or by means of a current of warm air (a water or steam bath will not succeed so well,) until the product shall weigh 14 ounces. To this must be added 1 ounces of rectified spirit. Should the roots not have been perfectly cleansed, the product must be digested with pure animal charcoal. *Liquor Taraxaci* resembles in colour pale Sherry, and possesses the acrid taste of the fresh root in an eminent degree. The dose is from one to three fluid drachms.—*Annals of Chemistry.*

